# Molecular Perspectives in Radioactive Iodine Theranostics: Current Redifferentiation Protocols for Mis-Differentiated Thyroid Cancer

**DOI:** 10.3390/jcm13133645

**Published:** 2024-06-21

**Authors:** Seza A. Gulec, Cristina Benites, Maria E. Cabanillas

**Affiliations:** 1 Miami Cancer Research Center, Miami, FL 33181, USA; 2 Herbert Wertheim College of Medicine, Florida International University, Miami, FL 33199, USA; 3 Kiran C Patel College of Allopathic Medicine, Nova Southeastern University, Fort Lauderdale, FL 33328, USA; 4 Department of Endocrine Neoplasia and Hormonal Disorders, The University of Texas MD Anderson Cancer Center, Houston, TX 77030, USA; mcabani@mdanderson.org

**Keywords:** thyroid cancer, differentiated thyroid cancer, mis-differentiated thyroid cancer, radioactive iodine refractory, radioactive iodine indifferent, redifferentiation, genomics, molecular theranostics, theranostic power, theranostic potential

## Abstract

Thyroid cancer molecular oncogenesis involves functional dedifferentiation. The initiating genomic alterations primarily affect the MAPK pathway signal transduction and generate an enhanced ERK output, which in turn results in suppression of the expression of transcription of the molecules of iodine metabolomics. The clinical end result of these molecular alterations is an attenuation in *theranostic power* of radioactive iodine (RAI). The utilization of RAI in systemic therapy of metastatic disease requires restoration of the functional differentiation. This concept has been accomplished by modulation of MAPK signaling. Objective responses have been demonstrated in metastatic disease settings. RAI-refractoriness in “differentiated thyroid cancers” remains a clinical problem despite optimized RAI administration protocols. Functional *mis-differentiation* and associated *RAI-indifference* are the underlying primary obstacles. MAPK pathway modulation offers a potential for reversal of RAI-indifference and combat refractoriness. This review presents the latest clinical experience and protocols for the redifferentiation of radioiodine-refractory *mis-differentiated* thyroid cancer, providing a comprehensive overview of the current protocols and intervention strategies used by leading institutions. Timing and techniques of imaging, thyrotropin (TSH) stimulation methods, and redifferentiation agents are presented. The efficacy and limitations of various approaches are discussed, providing an overview of the advantages and disadvantages associated with each of the protocols.

## 1. Introduction

The *theranostic potential* of radioactive iodine (RAI) was first conceptualized by Saul Hertz in 1936. He started preclinical experiments in 1937 and treated the first Graves patient in 1941 [1,2]. Hertz built the scientific framework of RAI theranostics, and it was also he who recognized the limits of *theranostic power* of RAI in thyroid cancer [3]. Following a report of an impressive clinical response to RAI in functional thyroid metastases, there was a quick incorporation of RAI theranostics in the management of “differentiated” thyroid cancer [4]. Many investigators of the time, however, were rather skeptical of RAI as a therapeutic agent, mainly because most thyroid cancers did not show full follicular differentiation with colloid formation, and therefore could not concentrate RAI [5,6]. Mid-century marked the dawn of nuclear medicine and theranostics, setting a new paradigm dominated by RAI, in the management of thyroid cancer [7,8]. The term “differentiated” thyroid cancer originally referred to a specific morphologic architecture and nuclear morphology. It was not intended to imply functional differentiation. “Differentiated” expression was introduced to the literature in the 1960s, to stress the significant differences in the clinical course of undifferentiated thyroid cancers and those showing papillary or follicular architectural patterns [9]. The nuance in the meaning of functional versus morphologic differentiation changed the philosophy of thyroid cancer treatment drastically. The term “differentiated” was adopted to indicate a functional attribute, thus leading to an assumption, a plausible one, that all “differentiated” thyroid cancers can effectively be treated with RAI. The term “differentiated” thyroid cancer (DTC) permeated the field, with numerous protocols developed by prominent physician scientists [4,10,11,12,13,14,15]. 

The DTC term survived over half a century and remained carved in all classical textbooks. Some also preferred the term “well-differentiated” thyroid cancer, which only added to the ambiguity. The term DTC is also present in the 2022 WHO classification of thyroid neoplasms [16]. Though the new classification contained appropriate changes reflecting tumor biology and histogenesis, it continues to propagate the *misuse* of the term “differentiated” in the context of function and theranostics. Functional *mis-differentiation* of papillary thyroid cancers was clearly demonstrated in a seminal paper published by the thyroid cancer genome atlas (TCGA) working group in 2014 [17]. The study elegantly demonstrated that there are two major driver mutations (BRAF and RAS). Other, less common, genomic alterations transcriptomically behaved similarly to BRAF and RAS (BRAF-like and RAS-like). The transcriptomic, proteomic, metabolomic, and phenomic consequences of the genomic events initiating and propagating thyroid cancer have been extensively studied within the last decade [18,19,20]. The data lead to a conclusion that the *theranostic power* of RAI is dependent on the full expression of genes of iodine metabolism, involved in uptake, organification, and transportation.

## 2. RAI-Refractoriness and RAI-Indifference

RAI-refractory thyroid cancer is a clinical definition indicating a poor or no objective response to RAI of so-called “differentiated thyroid cancers”. There are three distinct mechanisms of refractoriness: First and foremost is RAI-indifference. RAI-indifference implies a lack of avidity or reactivity in engaging RAI processing by the malignant tissue. Simply, the malignant tissue is not metabolically equipped to take up and/or process RAI as readily as the normal thyroid tissue. Due to the depressed iodine transcriptome, depending on the genomic signature, the *mis-differentiated* cancers exhibit variable degrees of *RAI-indifference.* The *theranostic power* of RAI, thus, is significantly diminished. The restoration of differentiation requires modulation of molecular pathways. Functional *mis-differentiation* primarily involves the MAPK-ERK signal transduction pathway. Most current strategies target the oncoprotein mutations in the MAPK-ERK pathway. The transcriptional, translational, and post-translational regulatory mechanisms involved and their impact on morphologic and functional differentiation have been largely characterized [18,19,20]. The role of the PI3K-mTOR pathway in thyroid oncogenesis is also well known. However, its contribution to functional *mis-differentiation* is not established as strongly as the MAPK-ERK pathway [21,22,23,24]. The role of receptor thyroid kinases as upstream regulators of MAPK-ERK signal transduction has also not been established for redifferentiation. The second mechanism for RAI-refractoriness involves a less-than-adequate radiation-absorbed dose delivery to the tumor. *RAI-insufficiency* may be a term to describe this mechanism. Most patients are treated with empirically determined administered activities. There is no linear correlation between administered activity and radiation-absorbed dose [11]. This approach could frequently lead to less-than-effective therapeutic radiation dose delivery. Though the importance of RAI dosimetry was recognized relatively early on and a number of dosimetric techniques and strategies have been developed over the years [12,13,25,26,27], the clinical impact of dosimetry is not fully appreciated and demonstrated. The third and last mechanism that may potentially contribute to RAI-refractoriness is RAI-resistance. RAI-resistance is a biologic response term and relates to the counteracting dynamics of cytolethal injury effects of beta radiation, cellular recovery potential, and its pace. The radiobiology of thyroid cancer and RAI is not included in this review.

## 3. Signaling Pathways and Functional Mis-Differentiation of Thyroid Cancers

**Figure 1:**
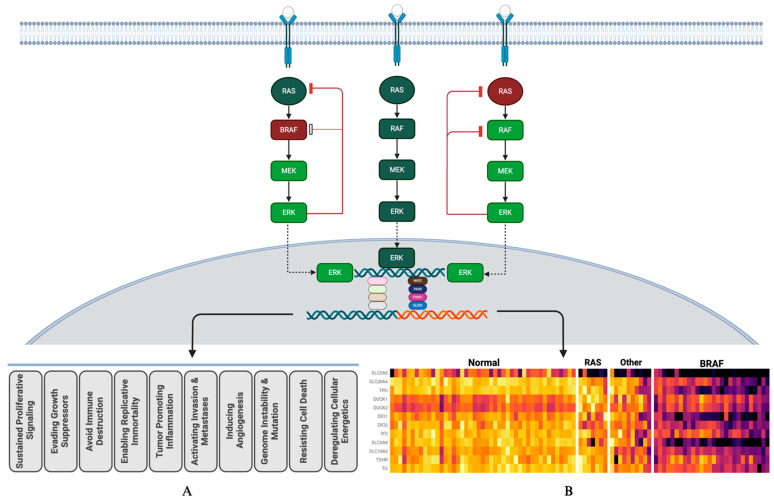
MAPK-ERK pathway and its ERK-mediated feedback regulation. A constitutive activation of the MAPK pathway results in enhanced ERK output. The intranuclear interactions of the ERK-mediated transcriptional program is rather complicated. There are two major directions for ERK-mediated transcriptional modifications. First is the generic oncogenic program producing the hallmarks of cancer phenotype: Panel (A). The second, the subject of this review, is the disruption of follicular functional differentiation via interruption of expression of genes associated with thyroid functional differentiation: Panel (B), a heat map depicting physiologic expression levels of iodine handling genes. From left to right: normal thyroid tissue, RAS-mutated cancers, non-RAS/non-BRAF cancers, and BRAF-mutated cancers. Darker colors indicate suppressed expression of genes. The lighter (brighter) colors of the iodine metabolic transcriptome under the “normal” column indicate regular expression of respective genes. ERK has feedback control over the MAPK pathway via RAS and RAF. For RAS-mutated cancers, ERK feedback inhibition works via both RAS and RAF. MEK-only inhibition is considered adequate for clinical redifferentiation. ERK feedback inhibition does not work with mutated BRAF. To achieve a clinically effective redifferentiation, MEK and BRAF combined inhibition is often required. Created with Biorender.com (https://app.biorender.com, Accessed 1 May 2024).

The signaling pathways start with activation of tyrosine kinase receptors (RTK) on the cell surface. There are a multitude of RTK constructs; these include VEGFR, PDGFR, IGFR, c-Kit, NTRK, ALK, RET, and MET. Receptor tyrosine kinases transmit an extracellular signal to intracellular target molecules. A sequential activation of the signal cascade(s) leads to oncogenic transformation including growth, proliferation, survival, and morphologic and functional mis-differentiation/dedifferentiation. The signal dysregulation and its oncopathophysiologic consequences are most comprehensively described for MAPK-ERK and, to a degree, PI3K-mTOR pathways. The existing data support a central role for constitutive activation of the MAPK-ERK pathway induced by oncoprotein mutations. The two most common mutations, BRAFv600E and RAS, are associated with a constitutive activation of the MAPK-ERK pathway, which results in an enhanced ERK output [18,28,29]. The ERK-mediated transcriptional program disrupts follicular morphologic differentiation and interrupts the expression of genes associated with thyroid functional differentiation (Figure 1). 

The expression of genes responsible for iodine uptake and metabolism is greatly reduced in BRAFV600E tumors, in contrast to RAS tumors, where the expression of these genes is preserved to a degree. MAPK-ERK pathway dysregulation and feedback control are different in BRAF- vs. RAS-initiated tumors. Those tumors driven by BRAFV600E do not respond to the negative feedback from ERK on RAF, resulting in higher MAPK-ERK signaling [30]. RAS-driven tumors, on the other hand, do respond to ERK feedback via blocking of RAF dimers. This results in a lower MAPK-ERK output. These feedback regulation dynamics affect MAPK-ERK output and degree of functional dedifferentiation as well as clinical redifferentiation strategies. The oncobiology, oncopathophysiology of tumor progression, and therapeutic responsiveness to MAPK-ERK pathway modulation strategies are categorically different for these two primary drivers [31]. The MAPK-ERK signal transduction can be decelerated, and ERK output-associated functional dedifferentiation can be mitigated. Current schemas of MAPK-ERK pathway modulation involve MEK inhibitors, BRAF inhibitors, and the combination of both [28,29,32]. BRAF-induced MAPK-ERK activation may not be controlled with MEK inhibition only, and may require combined MEK and BRAF inhibition, whereas the MAPK-ERK activation can be modulated with MEK inhibition alone more readily in RAS-mutation-initiated cancers [33,34,35] (Figure 1).

**Figure 2:**
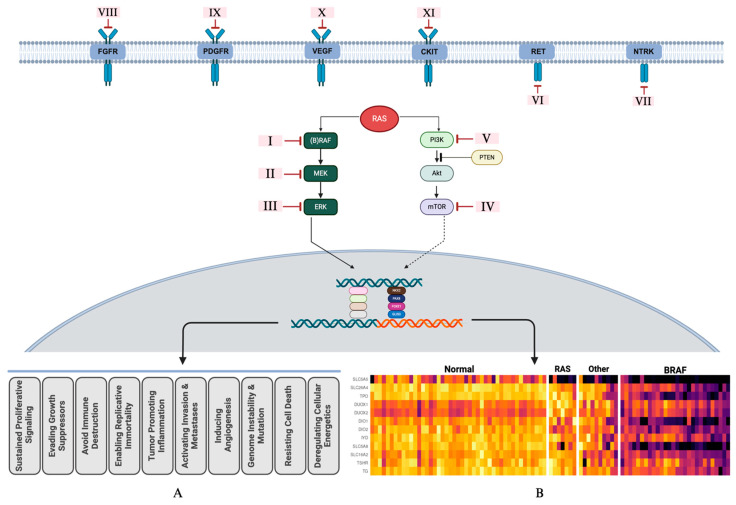
Signal transduction pathways leading to generic phenotypic expressions of oncogenesis (A) and specific iodine transcriptomic expression (B). Panel (B) is a heat map depicting physiologic expression levels of iodine handling genes. From left to right: normal thyroid tissue, RAS-mutated cancers, non-RAS/non-BRAF cancers, and BRAF-mutated cancers. Darker colors indicate suppressed expression of genes. The lighter (brighter) colors of the iodine metabolic transcriptome under the “normal” column indicate regular expression of respective genes. The MAPK-ERK pathway is well established and studied for effective modulation towards redifferentiation. The PI3K-mTOR pathway’s role in functional dedifferentiation and redifferentiation interventions are under investigation. **I**–**III**: MAPK-ERK pathway interventions. BRAF inhibitors: dabrafenib, vemurafenib, and encorafenib; MEK inhibitors: trametinib, cobimetinib, binimetinib, and selumetinib. IV–V: PI3K-mTOR pathway interventions. mTOR inhibitor: everolimus; PI3K inhibitor: copanlisib. **VI**–**VII**: RTK fusion oncoprotein interventions. RET fusion inhibitors: selpercatinib and pralsetinib; NTRK fusion inhibitors: larotrectinib and entrectinib. **VIII**–**XI**: RTK interventions. Multikinase inhibitors: lenvatinib: FGFR, PDGFR, VEGFR, and C-KIT; sorafenib: PDGFR, VEGFR, and C-KIT. Cabozantinib: MET and VEGFR2. The RAS molecule is activated by multiple TKRs and serves as a common inducting molecule for downstream signaling. Further specifics, including FDA approval status, of the therapeutic agents listed in this figure are listed in Table 1. Created with Biorender.com (https://app.biorender.com, Accessed 1 May 2024).

In vitro data suggest that a dual targeting of the MAPK-ERK and PI3K-mTOR pathways may also enhance the redifferentiation effect [21,22,23,24]. Clinical studies to investigate the role of PI3K-mTOR in functional dedifferentiation are underway. The role for multi-kinase inhibitors in redifferentiation is not clear at this stage. Multiple mechanisms and interactions amongst different pathways pose clinical challenges in designing effective and sustained redifferentiation protocols (Figure 2).

## 4. Clinical Protocols

### 4.1. Published Studies

The first successful clinical demonstration of redifferentiation with MAPK-ERK interference in RAI-indifferent cancer was published by Ho et al. in 2013 [38]. This was a Phase 2 study with 20 evaluable patients. Confirmation of metastatic disease was performed by CT and ^18^F-FDG-PET/CT. Molecular classification of the disease was documented, but both BRAF and RAS tumors were treated the same. Documentation/definition of RAI-refractory disease was made by I-124 PET/CT, 6 mCi. For thyroid stimulating hormone (TSH) stimulation, the rhTSH protocol was used. The redifferentiation strategy was selumetinib, a MEK inhibitor, at the dose of 75 mg po bid × 4 weeks. Demonstration of redifferentiation was made by I-124 PET/CT, 6 mCi. A dosimetry-guided I-131 treatment protocol was applied aiming for a minimum of 2000 cGy absorbed dose, with an administered activity of up to 300 mCi. In total, 12/20 (60%) of the patients demonstrated image evidence of redifferentiation. Evaluation of response was performed by CT or MRI, according to Response Evaluation Criteria in Solid Tumors (RECIST) and TSH/Thyroglobulin/Thyroglobulin antibody measurements 2 to 6 months after radioactive iodine therapy. This study led to a clinical trial comparing efficacy of adjuvant RAI treatment with or without MEK inhibition. The study failed to demonstrate a difference due to non-discrimination of BRAF and RAS categories [30]. MEK redifferentiation alone was later found to be inadequate for BRAF tumors. An important consideration in this study is the 6 mCi I-124 activity used for PET/CT imaging. A 6 mCi activity certainly enhances the theranostic power of I-124. Previous clinical experience has demonstrated that 1–2 mCi administered activity of I-124 was adequate to detect remnant tissue, thus may be considered to have 100% theranostic power/efficacy. 

The next structured redifferentiation study was published by Rothenberg et al. in March 2015, involving 10 evaluable patients [31]. Confirmation of metastatic disease was performed by CT and ultrasound. Molecular classification of the disease was documented, and only patients with BRAF tumors were treated. Documentation/definition of RAI-refractory disease was made by I-131 whole-body scan (WBS) (within 14 months of protocol treatments with administered activities of 2–4 mCi diagnostic or more than 29 mCi therapeutic). For TSH stimulation, the rhTSH protocol was used. The redifferentiation strategy was dabrafenib (a BRAF inhibitor) at the dose of 150 mg po bid x 4 weeks. Demonstration of redifferentiation was made by I-131 whole-body imaging, 4 mCi, on week 3 of protocol treatment. A formal dosimetry-guided I-131 treatment protocol was not applied. Those patients who showed radioactive iodine uptake on 4 mCi diagnostic imaging continued the protocol treatment and received 150 mCi of iodine 131 on week 5 (for patients who did not show I-131 uptake, no further treatment was given). Evaluation of response was performed by CT, according to RECIST and TSH/Thyroglobulin/Thyroglobulin antibody measurements 3 months after radioactive iodine therapy. 

Dunn et al. designed a Phase 2 trial using vemurafenib treatment in patients with BRAF-positive cancers [34]. The basic design of the study, including I-124 imaging and dosimetry as well as dosimetry-guided I-131 treatment, was similar to that of Ho et al. The image evidence of redifferentiation rate was 6/10 (60%). This study also demonstrated that BRAF inhibition with vemurafenib was associated with increased thyroid differentiation gene expression (improved thyroid differentiation score, TDS). 

Weber et al. studied 20 patients, 6 with BRAF and 14 with non-BRAF mutations, who received dabrafenib and trametinib combination and trametinib-alone redifferentiation treatments, respectively [36]. Documentation/definition of RAI-refractory disease as well as response to treatment evaluation was performed by using 4 mCi I-123 imaging and 1 mCi I-124 PET/CT imaging and dosimetry. TSH stimulation was performed using the rhTSH protocol. A dosimetry-guided I-131 treatment protocol was applied aiming for a minimum of 2000 cGy absorbed dose, with a mean administered activity of 300 mCi. In total, 7/20 (35%) of the patients demonstrated image evidence of redifferentiation. 

Leboulleux et al. published their experience in two multicenter trials led by Institute Gustave Roussy in 2023 [37,39]. Twenty-one evaluable patients with the BRAF mutation were treated with a dabrafenib (150 mg po bid) and trametinib (2 mg po qd) combination, and ten evaluable patients with RAS mutation were treated with trametinib alone (2 mg po qd) for 6 weeks. Documentation/definition of RAI-refractory disease was made by progression of the disease demonstrated by RECIST criteria on CT images, 12 months after an RAI treatment with a negative post-treatment whole-body scan (WBS). Patients underwent I-131 WBS, 5 mCi, for evaluation for RAI uptake. TSH stimulation was performed with the rhTSH protocol. Demonstration of/evaluation for redifferentiation was made by RAI imaging I-131 WBS, 5 mCi, and post-treatment I-131 WBS, 150 mCi. There was no formal dosimetry. All patients received a 150 mCi I-131 therapy dose on week 5 of redifferentiation protocol treatment. Evaluation of response was performed by CT, according to RECIST and TSH/Thyroglobulin/Thyroglobulin antibody measurements 1, 3, and 6 months after radioactive iodine therapy. A favorable objective imaging response was reported in 10/16 (63%) of the BRAF patients and 7/11 (66%) of the RAS patients. Though this trial was structured well, a consideration for future studies could be the use of dosimetry and addressing the potential stunning effect of 5 mCi I-131 to enhance the reliability of dose–response correlation. Table 2 provides a summary of the published redifferentiation studies. Response to therapy is addressed by image evidence of RAI uptake improvement. RECIST responses are not presented, as none of the published studies have provided absorbed dose correlations.

### 4.2. Clinical Trials in Progress

Following the initial promising results, a second wave of trials were designed with expansion of target oncoproteins to include RTKs and PI3K pathways as well as refined clinical protocols strengthened with dosimetric evaluations.

Ho et al. initiated a Phase 1 trial in July 2020, including only patients with BRAF-mutated tumors [40]. Documentation/definition of RAI-refractory disease is made using previously established imaging/dosimetry protocol with rhTSH and 6 mCi I-124 PET/CT. The redifferentiation strategy is vemurafenib (a BRAF inhibitor) and copanlisib (a PIK3 inhibitor). A vemurafenib–copanlisib combination is administered following a dose escalation protocol. The I-131 treatment is given combining a lesional dosimetry aiming for a minimum of 2000 cGy lesional absorbed dose and an administered activity limited to maximum tolerable activity (MTA). This Phase I safety/dose escalation study for combined use of vemurafenib and copanlisib evaluates redifferentiation by imaging in 1–2 weeks using 6 mCi I-124 PET/CT. Those patients demonstrating redifferentiation permitting an absorbed dose above 2000 cGy are treated with I-131. This is a comprehensive protocol requiring a high level of interdisciplinary coordination and patient cooperation.

Scott et al. initiated a multicenter trial sponsored by the Olivia Newton-John Cancer Research Institute [41]. This is a Phase 2 multicenter clinical trial for BRAF- and RAS-mutated thyroid cancers. Confirmation of metastatic disease is performed by 18F-FDG-PET and I-124 PET/CT. Molecular classification of the disease is documented. BRAF tumors are treated with dabrafenib (BRAF inhibitor) 75 mg and 2 mg po bid plus trametinib (MEK inhibitor) 75 mg and 2 mg po bid × 4 weeks, and RAS tumors are treated with trametinib 75 mg and 2 mg po bid × 4 weeks. Documentation/definition of RAI-refractory disease is made by I-124 PET/CT, 1 mCi. For TSH stimulation, the thyroid hormone withdrawal protocol is used. Demonstration of redifferentiation is made by I-124 PET/CT, 1 mCi. A dosimetry-guided I-131 treatment protocol is applied aiming for a minimum of 2000 cGy absorbed dose, with a fixed I-131 treatment activity of 150 mCi. If the estimated dose is <2000 cGy, patients are not treated. A dosimetric evaluation is performed to establish the dose–response relationship at the time of follow-up evaluations at 3 and 6 months. Evaluation of response is performed by CT (RECIST criteria) and FDG-PET/CT (PERCIST criteria) and TSH/Thyroglobulin/Thyroglobulin antibody measurements 3 and 6 months after radioactive iodine therapy. This is a highly refined protocol integrating I-124 imaging and dosimetry. A 1 mCi activity for I-124 imaging/dosimetry has a strong rationale behind it and may set a standard from logistics and cost perspectives.

Peiling Yang et al. initiated a single institutional trial sponsored by National University Hospital of Singapore [42]. This is a Phase 2 clinical trial for BRAF- and RAS-mutated thyroid cancers. BRAF tumors are treated with dabrafenib (BRAF inhibitor) plus trametinib (MEK inhibitor), and RAS tumors are treated with trametinib only. Documentation/definition of RAI-refractory disease is made by I-124 PET/CT, 2 mCi. For TSH stimulation, TSH withdrawal protocol is used. A dosimetry-guided I-131 treatment protocol is applied aiming for a minimum of 2000 cGy absorbed dose, with a maximum I-131 treatment activity of 300 mCi. If the estimated dose is <2000 cGy, patients are not treated. Evaluation of response is performed by CT (RECIST criteria) and FDG-PET/CT (PERCIST criteria) and TSH/Thyroglobulin/Thyroglobulin antibody measurements in 3 and 6 months after radioactive iodine therapy. 

Fendler et al. have an active Phase 2 clinical trial which essentially is the continuation of the study published previously by Weber [43]. Patients are grouped as BRAF-mutated and BRAF-wild with other mutations. Patients in the BRAF group receive a dabrafenib + trametinib combination, and those with non-BRAF mutations receive trametinib alone. Documentation/definition of RAI-refractory disease as well as response to treatment evaluation were performed by using 4 mCi I-123 imaging and 1 mCi I-124 PET/CT imaging and dosimetry. TSH stimulation was performed using the rhTSH protocol. A dosimetry-guided I-131 treatment protocol is utilized aiming for a minimum of 2000 cGy absorbed dose, with a mean administered activity of 300 mCi. 

Kapitejin et al. designed a single institutional trial using lenvatinib, a multi-receptor tyrosine kinase inhibitor, as a redifferentiation agent [44]. Patients undergo 1 mCi I-124 PET/CT for baseline assessment of RAI-refractory disease. The patients are treated with lenvatinib for a total of 12 weeks. After 6- and 12-week treatment, patients undergo 1 mCi I-124 PET/CT dosimetry to evaluate the redifferentiation effect with estimation of absorbed dose to the lesions and maximum tolerable activity. Patients undergo subsequent I-131 therapy if a clinically meaningful lesion dose is expected, and toxicity is deemed acceptable. For all patients eligible for I-131 therapy, lenvatinib is discontinued prior to administration of I-131, and intra-therapeutic I-131 SPECT dosimetry is performed for dose verification. Patients who are not eligible for I-131 therapy continue lenvatinib treatment at the discretion of the treating physician. Biopsies are performed at baseline and after 6-week lenvatinib treatment to evaluate alterations at the transcriptional and translational level in sampled tumor lesions. The response to treatment evaluations includes F-18 FDG PET/CT and thyroglobulin (Tg) levels.

Wirth et al., led by MGH/Harvard, initiated a Phase 2 multicenter trial using selpercatinib (a RET inhibitor) for RET (fusion)-mutated thyroid cancers [45]. Documentation/definition of RAI-refractory disease is made by an I-131 whole-body scan (WBS) (within 14 months of protocol treatments with administered activities of 2–4 mCi diagnostic or therapeutic, more than 29 mCi I-131, and TSH induction by rhTSH). The redifferentiation strategy is selpercatinib (a RET inhibitor) twice daily × 4 weeks. A second course of selpercatinib is administered if the participant is demonstrating clinical benefit to the initial course of selpercatinib and deemed clinically appropriate by the treating investigator. Demonstration of redifferentiation is made by I-131 whole-body imaging, 4 mCi, on week 3 of protocol treatment. A formal dosimetry-guided I-131 treatment protocol is not applied. Those patients who showed radioactive iodine uptake on 4 mCi diagnostic imaging continued the protocol treatment and received 150 mCi of iodine 131 on week 5 (for patients who did not show I-131 uptake, no RAI treatment was given). Evaluation of response was performed by CT, according to RECIST and TSH/Thyroglobulin/Thyroglobulin antibody measurements 6 months after radioactive iodine therapy. The redifferentiation response is evaluated at 5 weeks. RAI is given if evidence of differentiation is seen with 4 mCi I-131 diagnostic study. Those patients demonstrating evidence of redifferentiation are treated with 150 mCi empiric I-131. The potential stunning effect of 4 mCi I-131 is of concern.

Laetsch et al., led by the Children’s Hospital of Philadelphia (CHOP) Multicenter (CHPP), initiated a Phase 2 multicenter trial using larotrectinib (an NTRK inhibitor) for NTRK (fusion)-mutated thyroid cancers [46]. The patients undergo a baseline RAI whole-body scan (WBS) to assess the RAI-avidity of their tumor per standard of care. Following approximately 4 weeks of targeted therapy, a WBS is repeated to determine whether the particular therapy achieved redifferentiation. Demonstration of redifferentiation is made by I-131 WB scan, 4 mCi. No dosimetry is used for selection of treatment activity. Patients are administered empiric activities based on tumor burden and toxicity considerations. Evaluation of response is performed by CT (RECIST criteria) and Thyroglobulin measurements 6 months after radioactive iodine therapy.

**Figure 3:**
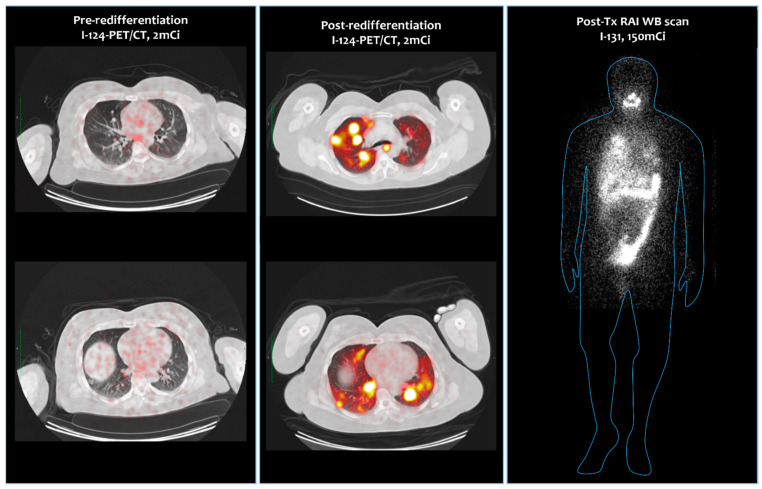
A successful redifferentiation of a BRAF (+) RAI-indifferent metastatic thyroid cancer with combined BRAF and MEK inhibition treatment. The first column of images on the left is the representative views of I-124 PET/CT obtained prior to redifferentiation treatment. The column in the middle demonstrates the views of I-124 PET/CT obtained after the redifferentiation treatment. The last column on the right is the post-treatment whole-body scan with 150 mCi activity (MCRC series).

Miami Cancer Research Center (MCRC) has a theranostic I-124 imaging and dosimetry protocol for evaluation of patients with thyroid cancer who are RAI-naïve or -refractory [47]. The patients are treated with RAI following established clinical guidelines. Absorbed dose calculations are performed using I-124 imaging and correlated with clinical objective responses. Patients deemed to have RAI-refractory disease follow a standard protocol. Confirmation of metastatic disease is performed by 18F-FDG-PET and I-124 PET/CT. Molecular classification of the disease is documented by full DNA sequencing with identification of the specific oncoprotein mutation. BRAF tumors are treated with dabrafenib (BRAF inhibitor) 75 mg and 2 mg po bid plus trametinib (MEK inhibitor) 75 mg and 2 mg po bid × 4 weeks, and RAS tumors are treated with trametinib 75 mg and 2 mg po bid × 4 weeks. The selection of treatment strategy for non-BRAF and non-RAS tumors is individually decided based on the type of oncoprotein mutation (oncogene-specific) and availability/choice of inhibitory pharmacologic agents (Table 3). Documentation/definition of RAI-refractory disease is made by I-124 PET/CT, 1 mCi. For TSH stimulation, the TSH withdrawal protocol is used. Demonstration of redifferentiation is made by I-124 PET/CT, 1–2 mCi. Theranostic dosimetry is not used for selection of treatment activity. The patients are treated empirically with 150–300 mCi I-131 based on tumor volume and toxicity considerations. A dosimetric evaluation is performed to establish the dose–response relationship at the time of follow-up evaluations in 6 months. Evaluation of response is performed by CT (RECIST criteria) and FDG-PET/CT (PERCIST criteria) and TSH/Thyroglobulin/Thyroglobulin antibody measurements 3 and 6 months after radioactive iodine therapy. Figure 3 demonstrates a successful redifferentiation obtained in a BRAF-mutated cancer with dabrafenib and trametinib combination. Table 3 Provides a summary of ongoing clinical trials.

## 5. Clinical Considerations with Redifferentiation Protocols

The protocol summaries provided in this review highlight the key clinical components and considerations in redifferentiation. The first step is the documentation/confirmation of the metastatic disease. Obviously, anatomic imaging modalities can demonstrate structural disease. FDG-PET/CT is a modality that may be used consistently and could provide both anatomic and functional information facilitating RECIST and PERCIST evaluations, respectively, and does not require iodinated contrast media. FDG uptake also has prognostic value [48]. Molecular classification of the disease is critical, as the core strategic decision is based on the mutational status of the tumors. Mutant BRAF and RAS activate MAPK signaling but do so to different degrees. The MAPK signaling flux induced by mutant RAS is dampened by negative feedback of activated ERK on RAF. This mechanism is disrupted with BRAF mutation. The mutated RAF is unresponsive to the negative feedback by activated ERK [20]. Currently, most protocols adopted treating BRAF tumors with BRAF inhibitor plus MEK inhibitor and RAS tumors with MEK inhibitor only. For BRAF tumors, BRAF inhibition alone or BRAF inhibition combined with PI3K pathway inhibition strategies are also viable options. The role for multi-kinase inhibitors such as lenvatinib, ERK-specific inhibitors, and PI3K pathway inhibitors are under investigation (Table 3). Definition/documentation of RAI-refractory disease is still unsettled [49]. Post-total thyroidectomy remnants are clearly visible with 2 mCi I-124 [50]. In fact, lesions under 0.5 cm in size that are not appreciable on CT/US imaging can be detected with I-124 at this activity level. The *theranostic power* of RAI for remnant tissue is 100% (physiologic standard *theranostic power*). RAI uptake in the malignant tissue is below 40% of the normal thyroid [4]. The range of suppression is dependent on the thyroid differentiation score (TDS) [51]. The protocol discussed above by Andrew Scott proposed 1 mCi activity for determination of the baseline and response to therapy evaluation with I-124 PET/CT. A 6 mCi activity used in MSKCC trials may not be necessary. If there is no visibly appreciable uptake in the known metastatic lesions, the tumor is RAI-indifferent and thus refractory. Dosimetry-guided treatment decisions on I-131 treatment can be performed by I-124 PET/CT, following a 4–8-week course of KI/MAPK/PI3K modulation. An arbitrary selection of 2000 cGy absorbed dose delivery to the index lesion(s) goal is a reasonable one but requires further clinical validation. Empiric clinical choices based on multiple disease- and patient-related considerations are also acceptable until data are complete and definitive. Objective response evaluations should definitely include FDG-PET/CT and RAI imaging based on the institutional choice of I-131 SPECT/CT or I-124 PET/CT. The relative merits and challenges for these modalities are outside the scope of this review. The TSH induction strategy is also an important consideration in a multi-stage intervention requiring complex coordination. The use of rhTSH for each diagnostic and therapeutic RAI intervention negates the need for thyroid hormone withdrawal and is preferable. Tight coordination is necessary to avoid logistical errors with time-sensitive use of rhTSH. If the thyroid hormone withdrawal protocol is used for TSH induction, to avoid a protracted hypothyroidism, a T3 replacement should be considered during the 4–8 weeks of KI-MAPK modulation. 

A very important consideration in the design, implementation, and outcome evaluations for redifferentiation is dosimetry. Reported correlations with *administered activity* vs. *RECIST/PERCIST responses* have a fundamental limitation. RECIST is an extremely meaningful endpoint in oncology. In the final analysis, what matters is whether the patients responded to RAI because there will be a significant impact on their treatment planning. What is problematic is the substitution of radiation absorbed dose (rad) with administered activity (mCi). A true dose–response evaluation most definitely requires dosimetric evaluation and relating RECIST to radiation absorbed dose. Administered activity (mCi) is not synonymous with radiation absorbed dose (Rad) as a measure for therapeutic power. A standard administered activity may deliver a wide range of absorbed doses and, at times, may be sub-therapeutic. Absorbed dose is what should be used for response evaluation. Response to treatment evaluations should compare rad (Gray in international units) dose to RECIST. A dosimetric evaluation is essential in establishing clinical response to redifferentiation treatment and post-redifferentiation RAI therapy.

**Figure 4:**
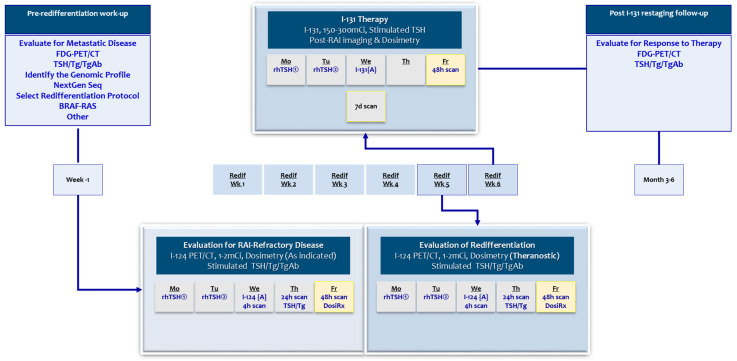
A flow chart for a comprehensive redifferentiation protocol. The pre-redifferentiation work-up includes (1) evaluation of the metastatic disease pattern and volume with functional/anatomic imaging, (2) Tg panel, (3) genomic and molecular profiling, and (4) determination of the clinically appropriate redifferentiation agent(s). Pre-redifferentiation work-up is completed 3–6 weeks prior to committing the redifferentiation drug therapy. The *evaluation for RAI-refractory disease* is performed by using I-124 PET/CT imaging. Preparation for imaging involves TSH stimulation. This can be performed with the rhTSH or withdrawal protocol. In the latter, the patients receive rhTSH injections on the first two days of the week block. I-124, 1–2 mCi, is administered on the third day. A complete image data set includes 3-day imaging for determination of cumulated activity (lesional and whole-body/bone marrow) and established MIRD voxel dosimetry. Each imaging time point also provides additional technical information (2–4 h imaging for calibration, 24 h imaging for conventional uptake determination, and 48 h is for SUV-based single-time-point predictive dosimetry). The patients then start the *redifferentiation drug therapy*. An *evaluation of redifferentiation* is performed in the fifth week block. This, essentially, is similar to I-124 imaging and the dosimetry protocol used prior to initiating the redifferentiation treatment. A decision to proceed with an anticipated RAI therapy is made at the end of this week. The TSH induction protocol is kept consistent for imaging and therapy interventions. With the rhTSH choice, the patients receive rhTSH injections on the first two days of the sixth week block. The *RAI therapy* is administered on the third day of the protocol week. The administered activity is determined by institutional preferences, typically in the range of 150–300 mCi I-131. The conventional post-treatment RAI scan, whole-body, and SPECT scan are performed on day 7 of the RAI administration. A 48 hour imaging is helpful for validation of pretreatment dosimetric evaluation. The restaging work-up is performed 3–6 m post-RAI-treatment and includes functional and anatomic imaging as well as the Tg panel.

Figure 4 is a flow chart/timeline for a suggested pre-treatment work-up, treatment protocol, and post-treatment follow-up.

## 6. Epilogue

Cellular oncogenic signal transduction pathway blockage, to revert the indifference to RAI prior to an RAI treatment in both the adjuvant treatment setting and the treatment for metastatic disease, is emerging as a new strategy. It can be regarded as a “neoadjuvant” intervention to render occult or overt disease responsive to RAI therapy. The theranostic power of RAI can be improved with new and evolving redifferentiation strategies.

A successful reversal of RAI-refractoriness at the molecular level is the first step in the improvement of RAI therapeutic efficacy. The second step involves the resolution of the problem of less-than-adequate radiation absorbed dose delivery into the tumor. The importance of RAI dosimetry is now more appreciated than before. Medical internal radiation dosimetry has made major strides in the past two decades. With the advent of high-resolution imaging systems and voxel dosimetry, a technically reliable and clinically feasible theranostic dosimetric technique can now be implemented. The last step in the RAI therapeutics is a comprehension of the intricacies of clinical response to RAI and its biologic modifiers. The concept of RAI-resistance/sensitivity needs to be better defined and understood. In short, RAI theranostics has entered a new paradigm to be built on a new clear foundation.

## 7. Conclusions

Our theranostic approaches for thyroid cancers have drastically changed parallel to our understanding of their oncobiology. Targeted therapies, modulating MAPK signaling pathway, and others effectively reverse the RAI-indifference encountered in *mis-differentiated* thyroid cancers and allow improved RAI theranostics. The translation of objective responses to clinical outcome measures are yet to be determined.

## Figures and Tables

**Table 1:** Current portfolio of targeted therapies for thyroid cancer classified based on their main category, FDA approval status, and clinical experience as a redifferentiation agent. FDA = Food and Drug Administration; RAI = radioactive iodine; DTC = differentiated thyroid cancer; MTC = medullary thyroid cancer; NSCLC = non-small cell lung cancer.

CATEGORY	AGENT	TARGET	FDA Approval	RAI Redifferentiation Report in Thyroid Cancer
TKR	Lenvatinib	MULTIKINASE	DTC	
	Sorafenib	MULTIKINASE	DTC	
	Cabozantinib	MULTIKINASE	DTC	
	Vandetanib	MULTIKINASE	MTC	
	Entrectinib	NTRK	DTC (NTRK)	
	Larotrectinib	NTRK	DTC (NTRK)	
	Repotrectinib	NTRK	NSCLC	
	Lorlatinib	ALK	NSCLC	
	Crizotinib	ALK	NSCLC	
	Alectinib	ALK	NSCLC	
	Selpercatinib	RET	DTC (RET)	In trial: NCT05668962
	Pralsetinib	RET	DTC (RET)	
MAPK|ERK	Dabrafenib	BRAF	BRAFv600 mutation	Published [31,36,37]
	Vemurafenib	BRAF	BRAFv600 mutation	Published [34]
	Encorafenib	BRAF	Colorectal cancer	
	Tavorafenib	BRAF(PAN)	Glioma	
	Selumetinib	MEK	Neurofibromatosis	Published [38]
	Trametinib	MEK	BRAFv600 mutation	Published [36,37,39]
	Ulixertinib	ERK	NOT APPROVED	
PI3K|mTOR	Copanlisib	PI3K	Lymphoma	In trial: NCT04462471
	Everolimus	mTOR	Neuroendocrine Tumors	

**Table 2:** Provides a summary of the published redifferentiation studies.

Lead Investigator, Year Publication Reference	Patients/Disease Redifferentiation Protocol	RAI Imaging, Dosimetry/Treat.	Redifferentiation Evidence by Imaging
Ho, 2013 [38]	20 9, BRAF, 5RAS, 6 Other Selumetinib	I-124, 6 mCi Lesional dosimetry D > 20 Gy, Max 300 mCi	12/20 (60%)
Rothenberg, 2015 [31]	10, BRAF Dabrafenib	I-131, 4 mCi No dosimetry 150 mCi Empiric	6/10 (60%)
Dunn, 2019 [34]	10, BRAF Vemurafenib	I-124, 6 mCi Lesional dosimetry D > 20 Gy, Max 300 mCi	6/10 (60%)
Weber, 2022 [37]	20 6, BRAF: Dabrafenib + Trametinib 14, Non-BRAF: Trametinib	I-123, 4 mCi & I-124 1 mCi Lesional dosimetry, MTA, mean 300 mCi	7/20 (35%)
Leboulleux, 2023 [38]	21, BRAF Dabrafenib +Trametinib	I-131, 5 mCi No dosimetry 150 mCi Empiric	10/16 (63%)
Leboulleux, 2023 [39]	10, RAS Trametinib	I-131, 5 mCi No dosimetry 150 mCi Empiric	7/11 (66%)

**Table 3:** Provides a summary of ongoing clinical trials.

Investigator, Trial Type, Trial Reference	Disease & Redifferentiation Protocol	RAI Imaging, Dosimetry	RAI Therapy
Ho, A. Phase I, MSKCC, New York, NY, US NCT04462471, recruitment ended	BRAF: vemurafenib + copanlisib	I-124, 6 mCi Lesional dosimetry	Dosimetry-Guided D > 20 Gy, Max 300 mCi
Scott A Phase II, AH, Melbourne, AU NCT05182931, recruitment active	BRAF: dabrafenib + trametinib	I-124, 1 mCi Lesional dosimetry	Dosimetry-Guided D > 20 Gy, Max 300 mCi
Peiling YS, Phase II, NUH, Singapore, SG NCT04554680, recruitment active	BRAF: dabrafenib + trametinib RAS: trametinib	I-124, 2 mCi Lesional dosimetry	Dosimetry-Guided D > 20 Gy, Max 300 mCi
Fendler, W. Phase II, EUH, Essen, DE NCT04619316, recruitment active	BRAF: dabrafenib + trametinib Non-BRAF: trametinib	I-123, 4 mCi + I-124 1mCi Lesional dosimetry	MTA dosimetry-guided
Kapiteijn, H, Phase II, Leiden, NL NCT04858867, recruitment closed	DTC: lenvatinib	I-124, 1 mCi Lesional dosimetry	Dosimetry-Guided D > 20 Gy, Max 200 mCi
Wirth, LJ, Phase II, MGH, Boston, MA, US NCT05668962, recruitment active	RET Fusion: selpercatinib	Post-RAI Tx imaging No Dosimetry	150 mCi Empiric
Laetsch, T, Phase II, CHOP, Philadelphia, PA, US NCT05783323, recruitment active	NTRK Fusion: larotrectinib	Post-RAI Tx imaging No Dosimetry	150 mCi Empiric
Gulec S. Phase II, MCRC, Miami, FL, US NCT06443866, recruitment active	Oncoprotein/RTK-targeted	I-124, 2 mCi Lesional dosimetry	150–300 mCi Empiric

## Glossary

Theranostics	Direct linking of diagnostic information to a therapeutic intervention. Theranostics describes a methodology in personalized medicine. In nuclear medicine, it refers to a radioactive pharmaceutical that is used to identify (diagnose) and to treat cancer.
Theranostic Power	Theranostic power defines the clinical performance of a theranostic agent or method, whereas theranostic potential is an abstract term indicating a potential of a diagnostic agent/technique/method to be a theranostic tool. The clinical performance of a theranostic tool is a function of biologic and technical determinants. The theranostic power of RAI in thyroid cancer is diminished due to suppressed iodine metabolic transcriptomics.
Differentiated Thyroid Cancer	Differentiated thyroid cancer refers to/encompasses thyroid cancers arising from thyroid follicular epithelial cells, excluding poorly differentiated and undifferentiated (anaplastic) varieties. Papillary thyroid cancer (PTC), follicular thyroid cancer (FTC), and oncocytic thyroid cancer (OTC) are considered differentiated. The term strictly implies a distinct morphologic differentiation. Iodine metabolic functionality of this group is highly variable. Metabolic function of these cancers is determined by their genomic and transcriptomic expressions. RAS-mutation-initiated cancers (follicular cancers and follicular variant of PTC) have diminished capacity for iodine processing. BRAF-mutation-initiated cancers (classic and tall cell variant PTC) have significantly depressed iodine metabolic function. Oncocytic cancers have completely different genomic constructs and have absent iodine processing capacity.
Mis-differentiated Thyroid Cancer	The term mis-differentiated refers to thyroid cancers with distinct morphologic differentiation, but functionally showing significant deviance in their iodine processing capacity, in the most part, missing the ability of full metabolic functionality.
RAI-Refractory	The term RAI-refractory denotes a lack of response to RAI therapy. A refractory state can be due to (1) inability to process RAI (RAI-indifference), (2) inadequate radiation absorbed dose delivery, or (3) radioresistance.
RAI-Indifference	RAI-indifference implies a lack of avidity or reactivity in engaging RAI processing by the malignant tissue. Simply, the malignant tissue is not metabolically equipped to take up and/or process RAI as readily as the normal thyroid tissue. Due to the depressed iodine transcriptome, depending on the genomic signature, the *mis-differentiated* cancers exhibit variable degrees of *RAI-indifference.*
RAI-Insufficiency	The mechanism for RAI-refractoriness involves a less-than-adequate radiation-absorbed dose delivery to the tumor. *RAI-insufficiency* may be a term to describe this mechanism.
RAI-Insensitivity (RAI-Resistance)	This is a distinct mechanism for RAI-refractoriness where the tumor, respective of its RAI avidity and adequate dose delivery, is biologically resistant to radiation effects. RAI-resistance is a biologic response term and relates to the counteracting dynamics of cytolethal injury effects of beta radiation and cellular repair mechanisms.
Redifferentiation	Oncoprotein-targeted molecular therapies that reverse the RAI-indifference and restore iodine metabolic functionality in mis-differentiated thyroid cancers.
